# Efficient Micropropagation Protocol for the Conservation of the Endangered *Aloe peglerae*, an Ornamental and Medicinal Species

**DOI:** 10.3390/plants9040506

**Published:** 2020-04-14

**Authors:** Nontobeko A. Hlatshwayo, Stephen O. Amoo, Joshua O. Olowoyo, Karel Doležal

**Affiliations:** 1 Agricultural Research Council—Vegetable and Ornamental Plants, Private Bag X293, Pretoria 0001, South Africa; HlatswayoN@arc.agric.za; 2 Department of Biology, Sefako Makgatho Health Sciences University, P. O. Box 139, Medunsa 0204, South Africa; joshua.olowoyo@smu.ac.za; 3 Laboratory of Growth Regulators & Department of Chemical Biology and Genetics, Centre of the Region Haná for Biotechnological and Agricultural Research, Faculty of Science, Palacký University & Institute of Experimental Botany AS CR, Šlechtitelů 11, CZ-78371 Olomouc, Czech Republic; karel.dolezal@upol.cz

**Keywords:** *Aloe*, conservation, cytokinin, in vitro propagation, plant tissue culture, shoot proliferation

## Abstract

A number of *Aloe* species are facing an extremely high risk of extinction due to habitat loss and over-exploitation for medicinal and ornamental trade. The last global assessment of *Aloe peglerae* Schönland (in 2003) ranked its global conservation status as ‘endangered’ with a decreasing population trend. In the National Red List of South African Plants, the extremely rapid decline of this species has resulted in its conservation status being elevated from ‘endangered’ to ‘critically endangered’ based on recent or new field information. This dramatic decline necessitates the development of a simple, rapid and efficient micropropagation protocol as a conservation measure. An in vitro propagation protocol was therefore established with the regeneration of 12 shoots per shoot-tip explant within 8 weeks using Murashige and Skoog (MS) medium supplemented with 2.5 µM *meta*-topolin riboside (an aromatic cytokinin). The rooting of the shoots with a 100% frequency on half-strength MS medium without any plant growth resulted in additional six shoots produced per cultured shoot. The resultant plantlets were successfully acclimatized with a 100% survival frequency after 6 weeks. Overall, the developed protocol can result in the production of 3906 transplantable shoots that are ready for rooting per annum from a single shoot-tip explant. It is simple and efficient for seedling production in the ex situ cultivation and conservation of the endangered *A*. *peglerae*.

## 1. Introduction

The succulent *Aloe* genus (Asphodelaceae) comprises of over 500 recognized species distributed throughout the world in different growth forms, ranging from very small shrubs to large trees [1,2]. Majority of the species are distributed on the African continent [3], especially in the southern and eastern regions, with high centers of diversity in South Africa (±155 *Aloe* species) and Madagascar (±145 *Aloe* species) [4]. According to Raimondo et al. [5], there are about 125 *Aloe* species indigenous to South Africa, many of which are known for their medicinal and/or ornamental values [2,6]. Medicinally, *Aloe* species are traditionally used in the treatment of injuries, infections, digestive ailments, inflammation, arthritis, insect bites and as a laxative, amongst others [1,2,3]. Thus, due to their increasing rarity and trade demand amongst other factors, all *Aloe* species with the exception of *Aloe vera* are included at least in Appendix II of the Convention on International Trade in Endangered Species of Wild Fauna and Flora [7] for their protection. 

Of the 125 indigenous South Africa *Aloe* species, Raimondo et al. [5] listed five species as ‘critically endangered’ in the National Red List of South African Plants while four species were listed as ‘endangered’ and 15 species ranked as ‘vulnerable’, based on the International Union of Conservation of Nature (IUCN) criteria. An updated National Red List of South African Plants however, indicated that there are now seven species ranked as ‘critically endangered’, five species listed as ‘endangered’, and 14 species considered to be ‘vulnerable’ based on the IUCN criteria [8]. Thus, it is very clear that a number of *Aloe* species are facing an extremely high risk of extinction in the wild, with a negative outlook. One of the species with a recent change in status in the National Red List of South African Plants, from being ‘endangered’ to ‘critically endangered’ based on recent or new field information is *Aloe peglerae* Schönland [8]. It is an endemic, ornamental and medicinal species used in the treatment of infections, burns, wound healing and as a laxative [9,10,11]. Based on its last global assessment in 2003, *Aloe peglerae* global conservation status is ranked as ‘endangered’, with a decreasing population trend [12]. It is threatened by illegal collection of mature plants and habitat loss due to urbanization [13,14]. Being a slow-growing species, its wild harvesting, even at very low levels, is unsustainable [14]. The ability of the known populations to recover from the ongoing, rapid population decline is hampered by high levels of seed harvesting for horticultural trade [13,14,15]. The extremely rapid decline of this species necessitates the implementation of effective conservation measures to ensure its survival and sustainable use. Its ex situ cultivation has been recommended as a conservation measure [13]. Availability of sufficient propagules/seedlings for large-scale cultivation in order to sustainably meet its growing demand is, however, another bottleneck. Amoo et al. [2] recommended the use of biotechnological tools such as micropropagation techniques as a conservation strategy for the sustainable use of threatened *Aloe* species or those in high demand. The aim of the current study was therefore to develop a simple, rapid and efficient micropropagation protocol for *Aloe peglerae*. To our knowledge, there was no such protocol prior to this report. 

## 2. Materials and Methods 

### 2.1. In Vitro Seed Germination and Explant Bulking

*Aloe peglerae* seeds purchased from Silverhill Seeds (Cape Town, South Africa) were surface-decontaminated using 70% ethanol for 60 s followed by the use of 3.5% sodium hypochlorite solution containing a few drops of Tween^®^ 20 [16] for 3 min. Following surface decontamination, the seeds were cultured on one-tenth strength Murashige and Skoog (MS) medium [17]. Bulking up of plant material for shoot proliferation experiments was achieved using in vitro raised shoot-tips cultured on MS medium supplemented with 30 g L^−1^ sucrose, 0.1 g L^−1^ myo-inositol, 7.5 µM *meta*-topolin (*m*T) and 2.5 µM indole butyric acid (IBA) [16]. All the growth media used were adjusted to a pH of 5.7 using NaOH or HCl. The media were solidified with 0.8% agar prior to autoclaving at 121 °C and 103 kPa for 20 min. Cultures were incubated in a growth room illuminated by cool, white fluorescent tubes (Osram^®^ L 36 W/640, 47 µmol m^−2^ s^−1^ light intensity) at 25 ± 2 °C and alternating 16:8 h light/dark.

### 2.2. Effect of Cytokinin Types and Concentrations on Shoot Multiplication

**Figure 1:**
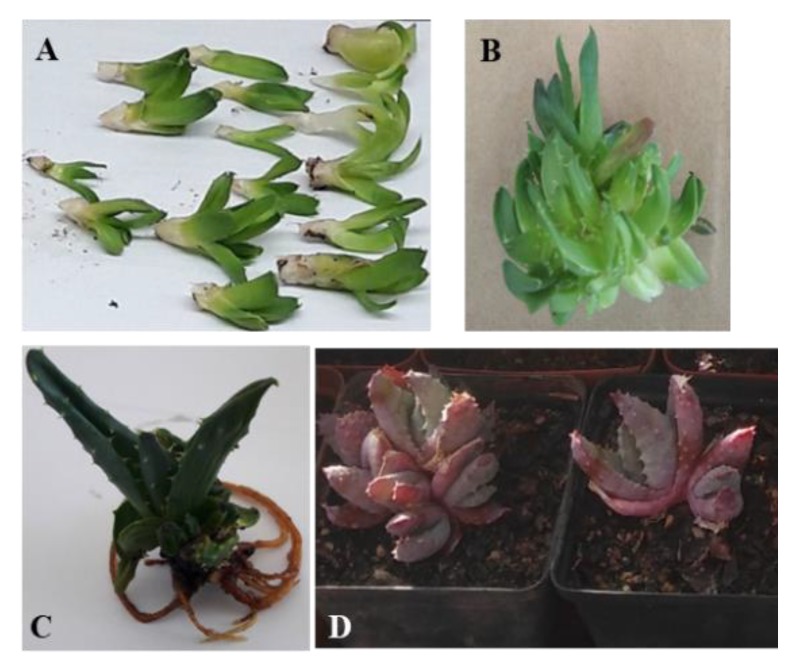
In vitro propagation of *Aloe peglerae*. (**a**) In vitro raised shoot-tip explants, (**b**) shoot proliferation, (**c**) rooting, and (**d**) acclimatization of in vitro regenerated plants.

After bulking sufficient shoot-tip explants (Figure 1), shoot proliferation experiments were conducted. Four different cytokinins; Kinetin, 6-benzyladenine (BA), *meta*-topolin (*m*T) and *meta*-topolin riboside (*m*TR), each at 2.5, 5.0, 7.5, 10.0 and 15.0 µM concentrations were evaluated for their shoot multiplication potential. Both *m*T and *m*TR were obtained from the Laboratory of Growth Regulators, Palacký University and Institute of Experimental Botany, Czech Republic. All other growth regulators used were purchased from Sigma-Aldrich (Johannesburg, South Africa). The MS medium without any plant growth regulator (PGR), which was the basal medium, was used as the control. The experiment was set up in a completely randomized design. One explant was cultured on 40 mL of medium contained in each jam jar. Each treatment had a total of 20 explants and the experiment was conducted twice. Cultures were incubated under the previously stated growth conditions above. Shoot proliferation parameters such as the total number of shoots per explant, number of shoots less than 1.0 cm in length, number of shoots greater than or equal to 1.0 cm in length, frequency of rooting, and shoot fresh weight were recorded after eight weeks of culture.

### 2.3. Effects of the Interaction of Auxin and Cytokinin on Shoot Proliferation

A completely randomized experiment involving four indole-butyric acid (IBA) concentrations (0.0, 1.0, 2.0, and 5.0 µM) in combination with 2.5 µM *m*TR was conducted. The choice of cytokinin type and concentration used at this stage was based on the results from the first shoot multiplication experiment. Each treatment had a total of 20 explants with one explant cultured on 40 mL of medium contained in each jam jar. The experiment was conducted twice. Culture incubation and data collection after eight weeks of incubation were the same as stated above. 

### 2.4. Rooting and Acclimatization

Regenerated shoots greater than 1.0 cm in length were carefully separated and cultured on half-strength MS medium supplemented with different concentrations (0.0, 1.0, 2.5 and 5.0 µM) of IBA or naphthalene acetic acid (NAA) in order to determine the effect of auxins on in vitro rooting. Cultures were incubated for six weeks under the same growth conditions stated previously. At the end of the six weeks culturing period, the following parameters were recorded: the number of roots per cultured shoot, rooting frequency, the length of the longest root, and number of additional shoots produced. Agar was carefully washed off the rooted shoots and the shoots planted in pots containing a 1:2 (v:v) mixture of sand and soil for acclimatization in a glasshouse. The survival frequencies of the acclimatized plantlets were recorded weekly for six weeks.

### 2.5. Extraction and Determination of Total Phenolic and Flavonoid Contents

Upon completion of each in vitro shoot proliferation experiment, the total phenolic and flavonoid contents of the resultant shoots were quantified. The plant materials were oven-dried at 50 °C before grinding to fine powders. Thereafter, the extraction method described by Makkar [18] was followed. Ground plant materials (0.2 g) were extracted with 10 mL of 50% aqueous methanol in a sonication bath for 20 min. Total phenolic content was quantified using the slightly modified Folin and Ciocalteu method as outlined by Fawole et al. [19]. The calibration curve was plotted using gallic acid as a standard and each determination done in triplicate. Total phenolic content was expressed in mg gallic acid equivalents (GAE) per g dry weight (DW). The flavonoid content was determined using the aluminum chloride colorimetric method described by Zhishen et al. [20]. Catechin was used as a standard for plotting the calibration curve and each determination done in triplicate. Flavonoid content was expressed in mg catechin equivalents (CE) per g DW.

### 2.6. Data Analysis

The data obtained were subjected to analysis of variance (ANOVA) using SPSS statistical software (version 16.0). Significant differences were established at *p* = 0.05 and the mean values were separated using Duncan’s Multiple Range Test.

## 3. Results

### 3.1. Shoot Proliferation

The effect of different cytokinin types and concentrations on adventitious shoot production, total phenolic and flavonoid contents are shown in Table 1. All the cytokinin types and concentrations, with the exception of 15 µM BA, *m*T and *m*TR as well as 10 µM BA, significantly increased shoot production when compared to the control (PGR-free) treatment. Shoot proliferation (Figure 1) decreased with an increase in cytokinin concentration. Overall, the MS medium supplemented with 2.5 µM *m*TR significantly gave the highest number of shoots per explant (12.17 shoots), which was six-fold of the shoot number produced in the control treatment. Similarly, an increase in cytokinin concentration gave a decrease in the number of shoots ≥ 1 cm in length. At the lowest cytokinin concentration, both *m*T and *m*TR treatments provided significantly highest number of shoots ≥ 1 cm in length when compared to all other treatments. The total phenolic and flavonoid contents of the regenerants varied significantly with cytokinin concentrations. Specifically, the total phenolic contents of all regenerants from *m*T (except 10 µM *m*T), *m*TR, and Kinetin treatments were significantly high when compared to the total phenolic content of regenerated shoots from the control treatment. On the other hand, none of the regenerants from BA treatments had significantly high total phenolic content, compared to the control treatment. Similarly, the flavonoid content of the regenerants from Kinetin (5 to 15 µM), *m*T (5, 10 and 15 µM), and *m*TR (2.5 to 7.5 µM) were significantly high compared to that of the control, whereas almost none of the regenerants obtained from BA treatments (except 2.5 µM) gave a significantly improved flavonoid content. Table 2 shows the effect of combining auxin concentrations with the optimum cytokinin concentration on shoot production, total phenolic and flavonoid contents. The inclusion of different auxin concentrations did not significantly increase shoot production, total phenolic and flavonoid contents.

### 3.2. Rooting and Acclimatization

Culturing regenerated shoots on half-strength MS growth medium containing no auxin gave the highest, desirable rooting frequency (Table 3). This same treatment resulted in a high number of roots per shoot, length of longest root, and number of additional shoots produced. On the other hand, the treatments with NAA gave low rooting frequency (Table 3). The number of roots produced per shoot, the length of the longest root and the number of additional shoots produced with all the NAA treatments were significantly low when compared to the control and some IBA treatments (Table 3). Increasing the concentration of IBA resulted in an increase in the number of roots per shoot and additional shoot production. However, none of the IBA treatments significantly improved rooting and additional shoot production when compared to the control. 

Following the acclimatization of the rooted plants (Figure 1), Table 4 shows their survival frequencies up to the sixth week. Only plants rooted in half-strength MS medium without auxin supplementation consistently gave a 100% survival frequency throughout the six weeks. All plantlets rooted with NAA treatments died after the first week while the survival frequencies of plantlets rooted with IBA treatments declined over the six weeks. 

## 4. Discussion

A simple, efficient in vitro propagation protocol was developed in this study using shoot-tip explants from in vitro raised seedling. The use of in vitro raised explants is advantageous for culture initiation as it reduces the common micropropagation challenge relating to culture contamination. Following culture initiation, the choice of cytokinin type and concentration is often a critical success factor in establishing an efficient micropropagation protocol for any plant species. Thus, we evaluated the effect of different concentrations of two commonly used cytokinins in plant tissue culture techniques (BA and Kinetin) and two naturally occurring hydroxylated derivatives of BA (*m*T and *m*TR). Many researchers have demonstrated several advantages associated with the use of *meta*-topolins in developing efficient propagation protocols for different plant species including *Aloe arborescens* Mill., *Aloe ferox* Mill. and *Aloe polyphylla* Pillans [16,21,22,23,24,25,26]. In the current study, both *m*T and *m*TR at the lowest concentration proved to be more efficient for in vitro shoot proliferation of *A*. *peglerae*. At equimolar optimal concentration (2.5 µM), the order of cytokinin efficiency for shoot proliferation in this study can be ranked as *m*TR > *m*T > Kinetin > BA (Table 1). 

In the micropropagation of *Artemisia amygdalina* Decne., a medicinally important species, Rasool et al. [27] reported a synergistic effect of auxins and cytokinins on shoot multiplication. Furthermore, Amoo and van Staden [28] indicated the potential amplifying effect of auxins on shoot proliferation when combined with the *meta*-topolins. Thus, we evaluated the effect of combining IBA concentrations with 2.5 µM *m*TR, being the optimum cytokinin concentration for multiple shoot production. Although there were increases in shoot proliferation with some auxin concentrations, these increases were not statistically significant (Table 2). Thus, our results indicated that further medium supplementation with IBA did not significantly improve shoot proliferation. Similarly, the combination of IBA or NAA with Zeatin or BA did not significantly improve shoot proliferation in *Aloe polyphylla* [29]. 

Culturing regenerated shoots on half-strength MS medium is an acclimatization step during the rooting phase. IBA treatments significantly enhanced rooting in comparison to the NAA treatments. (Table 3). IBA is known to be capable of slowly releasing indole-3-acetic acid (IAA), a more easily metabolized auxin whereas the stability and persistence of NAA in plant tissues can result in root growth inhibition [30]. This stability and persistence of NAA in the plant tissues might also alter or tilt the auxin:cytokinin ratio in the plant tissue to such a point that the ratio becomes less favorable for additional shoot production as observed with the NAA treatments. On the other hand, the use of half-strength medium alone was more favorable for rooting as it gave high rooting frequency, number of roots, number of additional shoots produced during rooting, and the longest root (Table 3). Similarly, Chukwujekwu et al. [29] reported best rooting results when regenerated *Aloe polyphylla* shoots were cultured on PGR-free MS medium. Root production without exogenous auxin application may be due to a sufficiently balanced production of endogenous auxin in the shoot apex, which is basipetally transported to the cut surface [31], favoring root growth and additional shoot production. 

The survival rate of in vitro produced plantlets under field conditions is one of the critical success factors in the development of an efficient micropropagation protocol [32]. The results from the current study indicate that in vitro rooting treatments can have an impact on the survival frequency of regenerated plants. The highest survival frequency was recorded with plants rooted in PGR-free medium whereas those rooted in medium containing NAA did not survive beyond the first week of acclimatization. Thus, rooting in PGR-free medium is cost-saving and efficient for high survival frequency of regenerated plantlets. 

As with other *Aloe* species, the bioactive secondary metabolites in *A. peglerae* are present mainly in the leaves [33,34,35]. Studies have shown that bioactive secondary metabolites can be influenced or altered by plant growth regulators applied during in vitro propagation stages [28,36,37]. In the current study, the choice of cytokinin and their concentrations significantly affected secondary metabolite production in *A. peglerae* leaf tissue, whereas the inclusion of auxin (IBA) concentrations did not markedly influence the secondary metabolite content of the regenerants. It is particularly noteworthy that the cytokinin treatment (2.5 µM *m*TR), which gave the best overall shoot production, also resulted in the regenerated shoots having higher secondary metabolite production when compared to the control treatment. Moreover, no morphological variation or abnormality was observed with the regenerated plants as the shoots were regenerated directly from the shoot-tips without an intervening callus phase and several subculture cycles that often predispose in vitro cultures to genetic variations [38,39,40]. 

## 5. Conclusions

Overall, this study established a simple, cost-effective, and highly efficient micropropagation protocol for the endangered *Aloe peglerae*. The superiority of aromatic cytokinins (*m*T and *m*TR) over the commonly used BA in adventitious shoot production was also demonstrated. High shoot multiplication rates were obtained at low cytokinin concentrations. Using a geometric progression that is based on the production of five transplantable shoots (shoots ≥ 1 cm in length) on medium containing 2.5 µM *m*TR and six possible multiplication cycles per annum, the developed multiplication protocol can result in the production of 3906 transplantable shoots that are ready for rooting per annum from a single shoot-tip explant. The rooting of these shoots using the developed protocol in this study can further result in the production of additional 19,530 shoots. Rooted plantlets were acclimatized with a high survival frequency, making this protocol efficient for seedling production in the ex situ cultivation and conservation of the endangered *A*. *peglerae*.

## Figures and Tables

**Table 1:** Effects of cytokinin types and concentrations on adventitious shoot production and secondary metabolite production of *Aloe peglerae* after 8 weeks of culture.

Cytokinin	Concentration (µM)	No. of Shoots Per Explant (n)	No. of Shoots		Individual Regenerated Shoot Fresh Weight (mg)	Rooting Frequency	Total Phenolic Concentration (mg GAE/g DW)	Flavonoid Concentration (mg CE/g DW)
			<1 cm in Length	≥1 cm in Length				
Control	0.0	2.00 ± 0.23 I ^1^	0.35 ± 0.15 h	1.65 ± 0.19 cd	943.01 ± 96.64 abcd	88.24%	13.41 ± 0.57 gh	0.97 ± 0.12 hij
BA	2.5	4.50 ± 0.70 efgh	2.85 ± 0.53 bcdef	1.65 ± 0.33 cd	850.71 ± 133.52 bcd	0%	13.98 ± 0.23 g	1.56 ± 0.27 ef
	5.0	4.79 ± 0.69 cdefg	3.16 ± 0.5 bcdef	1.63 ± 0.38 cd	702.93 ± 140.85 bcdef	5.26%	12.06 ± 0.06 i	1.07 ± 0.34 hij
	7.5	4.58 ± 0.53 defgh	2.84 ± 0.37 bcdef	1.74 ± 0.29 bcd	1243.70 ± 162.55 a	0%	11.93 ± 0.19 i	0.76 ± 0.11 j
	10.0	3.50 ± 0.44 fghi	2.00 ± 0.33 efg	1.50 ± 0.27 d	858.72 ± 115.07 bcd	0%	12.22 ± 0.33 i	0.90 ± 0.05 hij
	15.0	2.39 ± 0.40 hi	1.06 ± 0.26 gh	1.33 ± 0.20 d	1272.60 ± 183.11 a	0%	12.79 ± 0.19 hi	1.18 ± 0.21 fghi
Kinetin	2.5	6.84 ± 0.55 cd	3.58 ± 0.38 bcd	3.26 ± 0.44 b	309.79 ± 44.30 f	78.95%	22.51 ± 0.15 a	1.29 ± 0.05 fgh
	5.0	6.40 ± 0.72 cde	3.30 ± 0.49 bcdef	3.10 ± 0.56 bc	401.86 ± 92.13 f	55%	22.55 ± 0.34 a	1.51 ± 0.01 fg
	7.5	5.55 ± 0.77 cdef	3.00 ± 0.41 bcdef	2.55 ± 0.52 bcd	549.98 ± 98.62 def	40%	20.24 ± 0.51 bc	2.55 ± 0.03 bc
	10.0	5.40 ± 0.53 cdefg	3.35 ± 0.36 bcdef	2.05 ± 0.37 bcd	425.94 ± 96.18 ef	60%	20.14 ± 0.58 c	2.18 ± 0.02 cd
	15.0	4.85 ± 0.51 cdefg	2.90 ± 0.50 bcdef	1.95 ± 0.35 bcd	442.42 ± 80.88 ef	15%	22.11 ± 0.28 a	2.18 ± 0.00 cd
*m*T	2.5	9.20 ± 1.10 b	3.75 ± 0.45 bc	5.45 ± 0.99 a	630.68 ± 85.61 cdef	15%	21.15 ± 0.36 b	0.91 ± 0.06 hij
	5.0	5.67 ± 0.78 cdef	3.50 ± 0.52 bcde	2.17 ± 0.41 bcd	911.37 ± 168.46 abcd	0%	16.29 ± 0.33 f	2.96 ± 0.07 a
	7.5	4.45 ± 0.63 efgh	2.60 ± 0.34 cdef	1.85 ± 0.41 bcd	816.85 ± 92.44 bcde	0%	19.23 ± 0.31 cd	1.05 ± 0.04 hij
	10.0	4.35 ± 0.42 efgh	2.55 ± 0.32 cdef	1.80 ± 0.27 bcd	997.63 ± 211.50 abc	0%	10.22 ± 0.07 j	2.25 ± 0.06 cd
	15.0	3.24 ± 0.48 ghi	1.88 ± 0.36 fg	1.35 ± 0.24 d	1036.50 ± 107.56 ab	0%	18.67 ± 0.23 d	1.98 ± 0.04 d
*m*TR	2.5	12.17 ± 1.27 a	7.44 ± 0.91 a	4.72 ± 0.84 a	328.11 ± 32.53 f	16.67%	18.95 ± 0.16 d	1.93 ± 0.06 de
	5.0	6.90 ± 0.64 c	4.15 ± 0.55 b	2.75 ± 0.43 bcd	562.80 ± 106.12 def	20%	19.40 ± 0.42 cd	2.70 ± 0.11 ab
	7.5	5.74 ± 0.67 cdef	3.63 ± 0.53 bc	2.10 ± 0.36 bcd	705.71 ± 117.17 bcdef	15.79%	16.18 ± 0.42 f	1.92 ± 0.06 de
	10.0	6.25 ± 0.89 cde	3.40 ± 0.39 bcdef	2.85 ± 0.58 bcd	582.63 ± 81.51 def	0%	19.46 ± 0.40 cd	1.13 ± 0.19 ghij
	15.0	3.85 ± 0.51 fghi	2.10 ± 0.29 defg	1.75 ± 0.35 bcd	659.72 ± 84.88 bcdef	10%	17.69 ± 0.16 e	0.77 ± 0.02 ij

^1^ Mean values within the same column followed by different letter(s) are significantly different (*p* ≤ 0.05) based on Duncan Multiple Range Test.

**Table 2:** Effects of combining auxin concentrations with optimum cytokinin concentration on shoot production and secondary metabolite production of *Aloe peglerae* after 8 weeks of culture.

Treatment	No. of Shoots Per Explant (n)	No. of Shoots		Individual Regenerated Shoot Fresh Weight (mg)	Total Phenolic Concentration (mg GAE/g DW)	Flavonoid Concentration (mg CE/g DW)
		<1 cm in Length	≥1 cm in Length			
2.5 µM *m*TR + 0.0 µM IBA	13.82 ± 1.63 ab ^1^	8.94 ± 1.14 a	4.88 ± 1.21 a	390.94 ± 61.57 a	11.83 ± 0.27 a	2.57 ± 0.07 ab
2.5 µM *m*TR + 1.0 µM IBA	10.69 ± 1.24 b	7.38 ± 1.27 a	3.31 ± 0.90 a	561.99 ± 222.40 a	13.39 ± 1.69 a	2.73 ± 0.08 a
2.5 µM *m*TR + 2.0 µM IBA	16.71 ± 2.67 a	9.78 ± 2.03 a	6.92 ± 2.22 a	429.92 ± 103.04 a	12.17 ± 0.74 a	2.27 ± 0.09 c
2.5 µM *m*TR + 5.0 µM IBA	14.94 ± 1.93 ab	7.56 ± 1.14 a	7.38 ± 1.31 a	537.52 ± 131.05 a	14.70 ± 0.48 a	2.35 ± 0.10 bc

^1^ Mean values within the same column followed by different letter(s) are significantly different (*p* ≤ 0.05) based on Duncan Multiple Range Test.

**Table 3:** Effects of auxin concentrations on rooting of regenerated *Aloe peglerae* shoots after 6 weeks of culture.

Auxin	Concentration (µM)	No. of RootsPer Shoot	Length of Longest Root (cm)	No. of Additional Shoots Produced Per Cultured Shoot	Rooting Frequency (%)
Control	0.0 µM	9.00 ± 1.12 a ^1^	2.46 ± 0.22 a	6.00 ± 0.76 a	100
IBA	1.0 µM	6.60 ± 1.35 a	1.90 ± 0.33 ab	3.85 ± 0.87 b	70
	2.5 µM	7.55 ± 1.67 a	1.97 ± 0.39 ab	3.35 ± 0.77 bc	60
	5.0 µM	10.05 ± 2.71 a	1.45 ± 0.28 bc	4.75 ± 1.06 ab	75
NAA	1.0 µM	1.75 ± 0.66 b	0.85 ± 0.27 cd	1.25 ± 0.34 d	40
	2.5 µM	0.90 ± 0.28 b	0.73 ± 0.23 cd	1.70 ± 0.41 cd	40
	5.0 µM	0.37 ± 0.21 b	0.30 ± 0.17 d	0.21 ± 0.12 d	15

^1^ Mean values within the same column followed by different letter(s) are significantly different (*p* ≤ 0.05) based on Duncan Multiple Range Test.

**Table 4:** Survival frequency of regenerated *Aloe peglerae* plantlets rooted with different auxin concentrations and acclimatized (*n* = 20) in a glasshouse.

Auxin Type Used for Rooting	Concentration (µM)	Survival Frequency (%)					
		Week 1	Week 2	Week 3	Week 4	Week 5	Week 6
Control	0.0 µM	100	100	100	100	100	100
IBA	1.0 µM	100	100	92	92	92	85
	2.5 µM	100	100	83	75	67	67
	5.0 µM	100	93	93	87	87	80
NAA	1.0 µM	100	0	0	0	0	0
	2.5 µM	100	0	0	0	0	0
	5.0 µM	100	0	0	0	0	0
